# A Case of Cotyledonoid Dissecting Leiomyoma With Associated Disseminated Peritoneal Leiomyomatosis: The Significance of Frozen Section in Identification of This Unusual Entity

**DOI:** 10.7759/cureus.55781

**Published:** 2024-03-08

**Authors:** Diksha Karki, Girishma Shrestha, Saurav L Joshi

**Affiliations:** 1 Pathology, Bhaktapur Cancer Hospital, Bhaktapur, NPL; 2 Pathology, Patan Academy of Health Sciences, Lalitpur, NPL

**Keywords:** ihc, frozen section, disseminated peritoneal leiomyomatosis, cotyledonoid dissecting leiomyoma, leiomyoma

## Abstract

Cotyledonoid dissecting leiomyoma (CDL) is a rare variant of uterine leiomyoma. The tumor is benign, but the appearance and growth pattern are unusual and alarming. Disseminated peritoneal leiomyomatosis (DPL) is another rare tumor that can mimic malignancy. The occurrence of these two tumors in a single case is even rarer and has not been found in the literature to the best of our knowledge. We report a case of CDL with DPL in a 43-year-old Nepalese woman who presented with abdominal pain and per vaginal bleeding. Ultrasound showed a heterogeneous hypoechoic mass of size 25.1 × 15.5 × 9.4 cm in the pelvic cavity. A CT scan of the abdomen and pelvis revealed an ill-defined, heterogeneously enhancing lesion in the pelvis around the uterine fundus with a peritoneal nodule. The intraoperative frozen section evaluated the peritoneal deposit to be benign. Due to the large size of the uterine mass, a total abdominal hysterectomy and a bilateral salpingo-oophorectomy were performed. Macroscopically, a large heterogeneous intramural and exophytic mass was observed, which, on histopathology and immunohistochemistry (IHC), revealed the benign smooth muscle origin of the tumor. In the seven-month follow-up period, no recurrence or any other related complications were found. It is important to recognize this rare variant of leiomyoma with the possibility of dissemination that can also happen in leiomyoma to prevent aggressive and inappropriate overdiagnosis and overtreatment. Whenever possible, it is advisable to perform a frozen section biopsy and IHC for the correct diagnosis.

## Introduction

Cotyledonoid dissecting leiomyoma (CDL) is a rare variant of leiomyoma that was first described in a series of four cases by Roth et al. [1]. This variant of leiomyoma is characterized by multinodularity with dissection of the myometrium along with exophytic extrauterine growth that looks like the cotyledon of the placenta [1]. Disseminated peritoneal leiomyomatosis (DPL) is another rare benign entity where multiple nodules comprising smooth muscle are found throughout the peritoneal cavity [2]. The unusual growth pattern can make the identification of this benign tumor challenging both clinically and radiologically, and histomorphology, along with the addition of immunohistochemistry (IHC) markers, can pave the path to the correct diagnosis. The literature search shows individual case reports of CDL and DPL, but the occurrence of these two rare tumors in the same patient has not been reported. To our knowledge, this is the first reported case from Nepal.

## Case presentation

**Figure 1:**
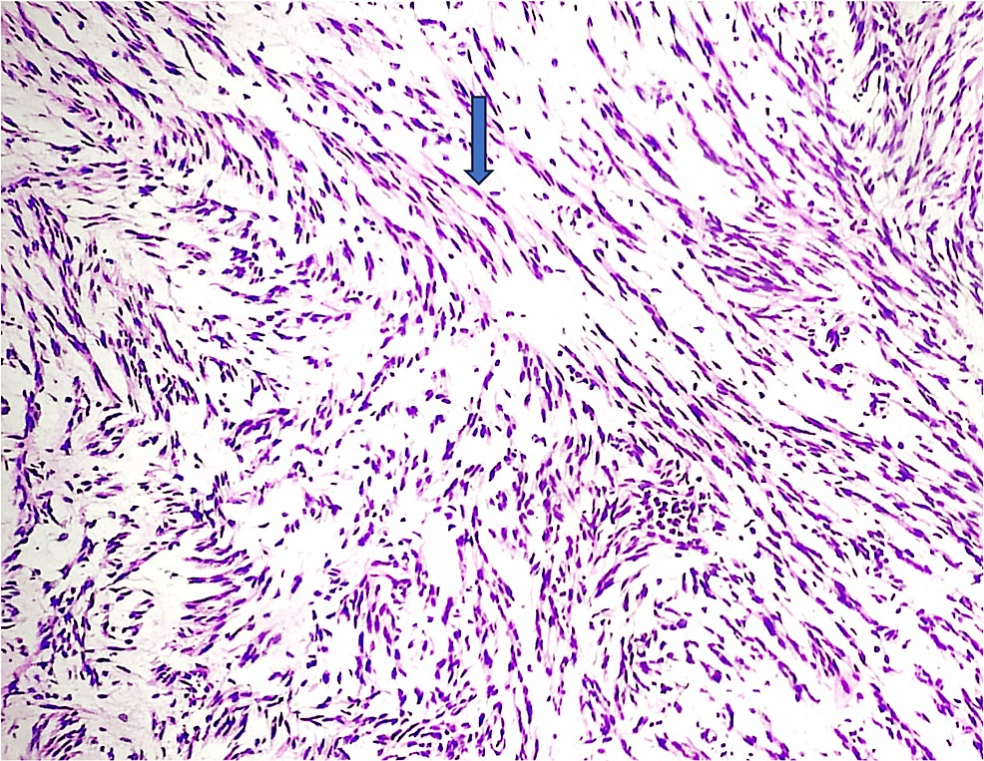
A frozen section biopsy from peritoneal deposits shows benign-looking spindle-shaped cells (shown in the blue arrow) with eosinophilic cytoplasm devoid of atypia, mitosis, and necrosis.

A 43-year-old Nepalese woman presented with a history of abdominal distension and pain for three months and per vaginal discharge for one month. Her complete blood counts were within normal limits. Ultrasonography of the abdomen and pelvis was done, which showed a pelvic mass measuring 25.1 × 15.5 × 9.4 cm with moderate echogenic fluid collection and probe tenderness. Her tumor marker CA125 was within normal limits. A CT scan of the abdomen and pelvis was performed, which showed an ill-defined heterogeneously enhancing lesion seen in the pelvis around the uterine fundus, encased within the peritoneal fluid collection without separate visualization of bilateral ovaries, and nodular enhancing thickening of the lining peritoneum, which was suspected to be bilateral ovarian malignancy with peritoneal metastasis. As the mass was suspicious of malignancy, the patient underwent a staging laparotomy, followed by a biopsy of the peritoneal deposit, right fallopian tube, and ovary, and was sent for a frozen section. A frozen section (Cryostat 6250, Dakewe) biopsy from peritoneal deposits showed benign-looking spindle-shaped cells with eosinophilic cytoplasm devoid of atypia (Figure 1).

**Figure 2:**
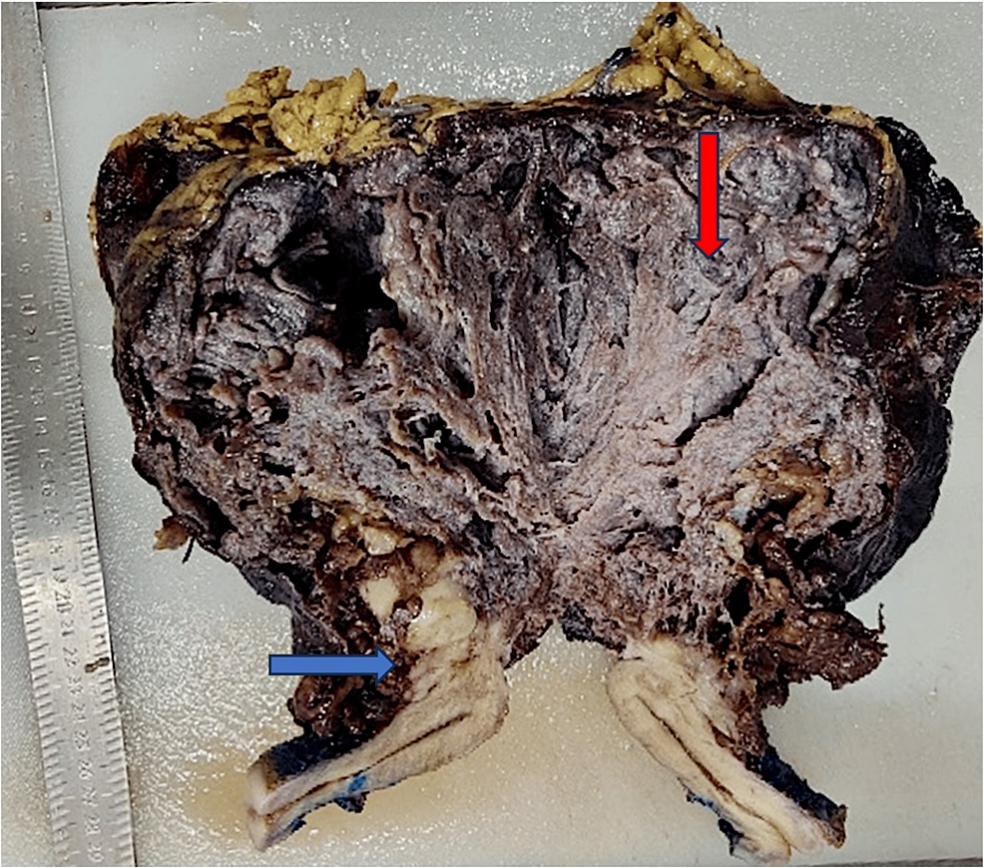
The cut surface shows an exophytic brown-colored solid cystic mass with interconnecting spaces (shown in the red arrow) and outer myometrial involvement. The myometrial component of mass has a multinodular appearance with a solid gray-white area (shown in the blue arrow).

Mitosis and necrosis were not identified. An intraoperative diagnosis of a benign spindle cell lesion was made. Sections from the right fallopian tube and ovary were negative for malignancy. Intraoperatively, a huge exophytic brown mass was seen arising from the posterior surface of the uterus, approximately measuring 20 × 15 × 10 cm, for which the patient underwent total abdominal hysterectomy, left salpingo-oophorectomy, pelvic peritoneal biopsy, and was sent to the histopathology lab. On gross examination, a mass of size 22 × 15 × 11 cm is present, arising from the posterior surface of the uterine serosa. The outer surface is irregular and dark brown. The cut surface shows an exophytic brown-colored solid cystic mass with interconnecting spaces with outer myometrial involvement. The myometrial component of mass has a multinodular appearance with a solid gray-white area (Figure 2).

**Figure 3:**
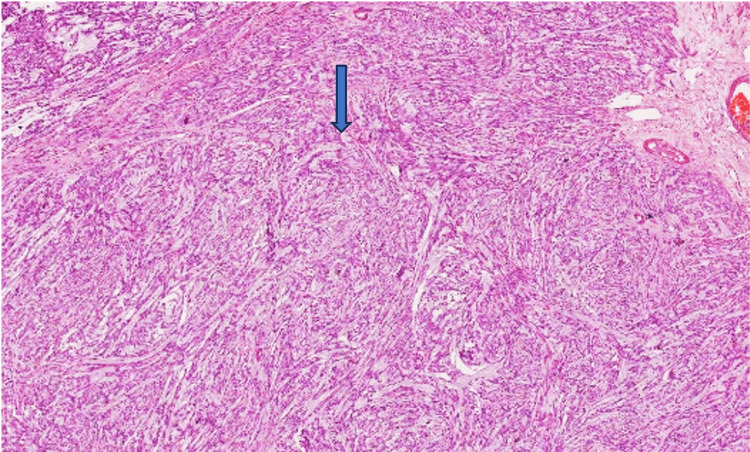
H&E-stained slide revealed spindle-shaped cells arranged in bundles and fascicles with a moderate amount of eosinophilic cytoplasm (shown in the blue arrow), uniformly elongated nuclei, a fine chromatin pattern, and inconspicuous nucleoli.

The boundary between the mass and the myometrium was unclear. No necrosis was identified grossly. The endometrium, cervix, and bilateral adnexa were grossly unremarkable. On microscopic examination, a H&E-stained slide revealed a multinodular pattern showing spindle-shaped cells arranged in bundles and fascicles with a moderate amount of eosinophilic cytoplasm, uniform elongated nuclei, a fine chromatin pattern, and inconspicuous nucleoli. Numerous congested and dilated vessels and edematous connective tissue between nodules were found (Figure 3).

**Figure 4:**
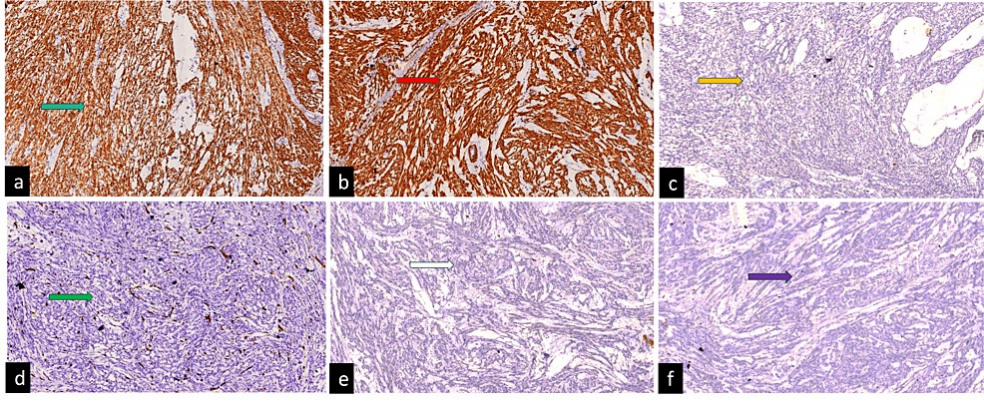
On IHC, tumor cells were positive for smooth muscle actin (a, green arrow) and desmin (b, red arrow). Ki67 labeling index was approximately <1% (c, yellow arrow), while they were negative for CD34 (d, green arrow), negative for DOG1 (e, white arrow), and negative for CK (AE1/AE3) (f, purple arrow). IHC, immunohistochemistry

Areas of hyalinization and hemorrhages were noted. The spindle cells are seen dissecting the myometrial fibers. No necrosis, atypia, or mitosis were identified. Sections examined from the multiple peritoneal deposits also show benign spindle-shaped cells having similar morphology and devoid of atypia, mitosis, and necrosis. Further, IHC was performed, and tumor cells were positive for smooth muscle actin (1A4, Dako) and desmin (D33, Dako) (Figure 4).

While negative for CD117 (c-kit, Dako), DOG1 (DOG1.1, Dako), CK (AE1/AE3, Dako), and CD34 (QBEnd10, Dako), Ki67 (MIB-1, Dako) labeling index was approximately <1%. Correlating the gross, histopathology, and IHC findings, a final diagnosis of CDL with DPL is rendered.

## Discussion

The CDL of the uterus, also known as “Sternberg tumor,” depicts an unusual growth pattern that can create a diagnostic dilemma [1]. The case presented exhibited an exophytic, huge mass arising from uterine serosa, which radiologically and intraoperatively suggested malignancy. In addition, the presence of multiple peritoneal nodules indicated a disseminated disease. In such circumstances, frozen section biopsy has proven to be a promising tool in determining the nature of the lesion, which will avoid unnecessary surgical procedures. The overall accuracy of the frozen section is 93.3%, which allows the surgeons to make appropriate intraoperative decisions [3]. In the present case, a frozen section biopsy from the peritoneal nodules showed spindle cells devoid of atypia and mitosis, suggesting they were benign, which prevented further radical dissection in this patient.

The unusual gross appearance of the tumor with multiple peritoneal nodules and leiomyosarcoma with peritoneal metastasis come under differentials [2,4,5]. However, the histopathological findings, including the absence of tumor coagulation necrosis, mitotic activity, cellular atypia, and a low ki67 index in the IHC, as seen in the current case, can rule out malignancy. The hormonal influence, intraperitoneal implants following morcellation of myoma, genetic factors, and metaplasia of submesothelial mesenchymal cells have been postulated for DPL [4,5]. None of such a history could be traced in the present case, except that she is of reproductive age.

After seven months of surgery, the patient is on regular follow-up, and no associated complications have been identified.

## Conclusions

CDL and DPL are rare tumors, and their occurrence in a single patient is an even rarer finding. Awareness of these tumors and recognition of their bizarre gross appearance are important for appropriate classification and to avoid unnecessary surgical procedures in patients. Whenever available, prompt frozen biopsy assessment and further application of IHC markers can pave the way for the correct diagnosis.
